# Biofilm Formation in *Acinetobacter Baumannii*: Genotype-Phenotype Correlation

**DOI:** 10.3390/molecules24101849

**Published:** 2019-05-14

**Authors:** Cheng-Hong Yang, Pai-Wei Su, Sin-Hua Moi, Li-Yeh Chuang

**Affiliations:** 1 Department of Electronic Engineering, National Kaohsiung University of Science and Technology, Kaohsiung 80778, Taiwan; chyang@cc.kuas.edu.tw (C.-H.Y.); moi9009@gmail.com (S.-H.M.); 2 Ph. D. Program in Biomedical Engineering, Kaohsiung Medical University, Kaohsiung 807, Taiwan; 3 Institute of Biotechnology and Chemical Engineering, I-Shou University, Kaohsiung 84001, Taiwan; czscientist@gmail.com; 4 General Education Center, Wenzao Ursuline University of Languages, Kaohsiung 807, Taiwan

**Keywords:** *Acinetobacter baumannii*, biofilm formation, antibiotic resistance, biofilm related gene

## Abstract

Strains of *Acinetobacter baumannii* are commensal and opportunistic pathogens that have emerged as problematic hospital pathogens due to its biofilm formation ability and multiple antibiotic resistances. The biofilm-associated pathogens usually exhibit dramatically decreased susceptibility to antibiotics. This study was aimed to investigate the correlation of biofilm-forming ability, antibiotic resistance and biofilm-related genes of 154 *A. baumannii* isolates which were collected from a teaching hospital in Taiwan. Biofilm-forming ability of the isolates was evaluated by crystal violet staining and observed by scanning electron microscopy. Antibiotic susceptibility was determined by disc diffusion method and minimum inhibitory concentration; the biofilm-related genes were screened by polymerase chain reaction. Results showed that among the 154 tested isolates, 15.6% of the clinical isolates were weak biofilm producers, while 32.5% and 45.4% of them possessed moderate and strong biofilm formation ability, respectively. The experimental results revealed that the multiple drug resistant isolates usually provided a higher biofilm formation. The prevalence of biofilm related genes including *bap*, *bla*_PER-1_, *csu*E and *omp*A among the isolated strains was 79.2%, 38.3%, 91.6%, and 68.8%, respectively. The results indicated that the antibiotic resistance, the formation of biofilm and the related genes were significantly correlated. The results of this study can effectively help to understand the antibiotic resistant mechanism and provides the valuable information to the screening, identification, diagnosis, treatment and control of clinical antibiotic-resistant pathogens.

## 1. Introduction

*Acinetobacter baumannii* is an important nosocomial pathogen that is responsible for a wide range of human infections [1,2]. Recently, the rapid development of multiple antibiotic resistance of *A. baumannii* has caused a serious problem for public health. The ability of biofilm formation contributes to *Acinetobacter* easily survive and transfer in the hospital environment, such as attached to various biotic and abiotic surfaces, e.g., vascular catheters, cerebrospinal fluid shunts or Foleys catheter [3,4]. Biofilms are assemblages of microorganisms, encased in a matrix, that function as a cooperative consortium to provide a protected mode for microorganisms and enhance resistance to various antibiotics [5]. Biofilm formation is a complex process employing many factors that include the aggregation substance, adhesion of collagen, expression of pili, and iron acquisition [6]. 

Among the several factors, the biofilm-associated protein encoded by the *bap* gene plays an important role in intercellular adhesion, accumulation of bacterial cells, and establishment of biofilm [7,8]. In the literature reports, the presence and expression of the *bla*_PER-1_ gene has been identified to encourage the clinical isolates of *A. baumannii* to form biofilm and adhere to respiratory epithelial cells [9,10,11]. The report extends previous observations by showing that the outer membrane protein A (*Omp*A) of *A. baumannii* 19606 plays a partial role in the development of robust biofilms on the plastic surface [10]. The ability of *A. baumannii* to form biofilms is also largely dependent on pili, which mediate attachment and biofilm formation. The genes are clustered together in the form of a *csu* operon, the products of which form a pilus-like bundle structure in *A. baumannii* [12]. Hence, the *csu*E gene also plays a major role in *A. baumannii* biofilm formation [13]. The bacterial and fungal biofilm formation has been suggested to decrease the diffusion of drugs through the bacterial and fungal cells and cause the persistence of clinical isolates under harsh environments with multidrug resistance [14,15,16,17]. 

However, it is currently unclear whether there is a quantitative correlation between biofilm formation and antibiotic resistance. In this study, 154 clinical *A. baumannii* isolates were investigated for their antibiotic susceptibility profile, biofilm formation and the biofilm related genes; we also analyzed the relationship between their phenotypes and genotypes.

The objective of this study was to determine the correlation between the ability of biofilm formation with distribution of biofilm related genes and antibiotic resistance phenotypes in the clinical isolates of *Acinetobacter baumannii*.

## 2. Results

### 2.1. Antibiotic Susceptibility Testing 

**Figure 1:**
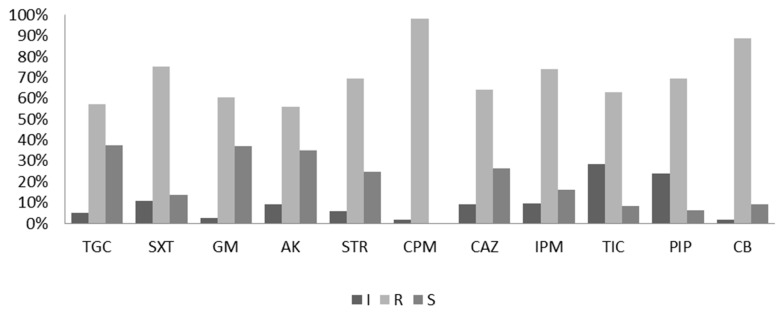
Antibiotic susceptibility test by the diffusion method. R, resistant; S, sensitive; I, intermediate. AK, amikacin; IPM, imipenem; TGC, tigecycline; CPM, cefepime; CAZ, ceftazidime; GM, gentamicin; TIC, ticarcillin; PIP, piperacillin; SXT, sulfamethoxazole/trimethoprim; CB, carbenicillin and STR, streptomycin.

The antibiotic susceptibility of the *A. baumannii* isolates was initially detected using the disk diffusion method [18]. Eleven antibiotic agents in the categories of aminoglycosides, cefepime, carbapenems, penicillins, folate pathway inhibitors, and tetracyclines were selected for the test. Among the 154 test isolates, resistance to cefepime (96.2%) was the most common, followed by resistances to carbenicillin (88.39%), sulfamethoxazole-trimethoprim (75.6%), ticarcillin (74.23%), piperacillin (69.75%), ceftazidime (69.7%), ciprofloxacin (65.8%), imipenem (65.67%), gentamicin (60.8%), tigecycline (57.6%), amikacin (56.17%), and streptomycin (56.17%), as shown in Figure 1. The results of the antibiotic susceptibility test revealed that the resistance rates of all strains were > 55% against all the tested antibiotics.

### 2.2. Minimum Inhibitory Concentration Determination

The minimal inhibitory concentrations (MICs) of the isolates against the 11 antibiotics were estimated using the broth dilution method [18]. According to the results obtained from the antibiotic susceptibility test, a total of 75 *A. baumannii* isolates were selected for the MIC determination. As shown in Table 1, less than 6% of the 75 isolates were slightly susceptible (S) to carbenicillin with an MIC of <16 µg/mL, 37% had intermediate sensitivity (I) against carbenicillin with an MIC of 16–32 µg/mL, and more than 56% of the strains had strong resistance (R) against carbenicillin with an MIC of ≥ 64 µg/mL. Against other antibiotics, isolates showed strong resistance as follows: 41% against gentamicin (MIC ≥ 16 µg/mL); 27% against amikacin (MIC ≥ 64 µg/mL); 32% against streptomycin (MIC ≥ 16 µg/mL); 59% against cefepime (MIC ≥ 16 µg/mL); 13% against ceftazidime (MIC ≥ 32 µg/mL); 28% against imipenem (MIC ≥ 8 µg/mL); 41% against ticarcillin (MIC ≥ 128 µg/mL); 43% against piperacillin (MIC ≥ 128 µg/mL); 56% against carbenicillin (MIC ≥ 64 µg/mL); 63% against trimethoprim-sulfamethoxazole (MIC ≥ 76 µg/mL); and 27% against tetracycline (MIC ≥ 16 µg/mL). Moreover, considering the antibiotic category, the highest strong resistance rates of *A. baumannii* isolates were found against aminoglycoside antibiotics (gentamicin, amikacin, and streptomycin) with resistance rates higher than 27%. Strong resistance was also found in the isolates against penicillins (ticarcillin, piperacillin, and carbenicillin) with resistance rates higher than 40%.

### 2.3. Relationship between Antibiotic Susceptibility and Biofilm Formation

The correlation between biofilm formation and resistance to the 11 antimicrobial agents in *A. baumannii* was analyzed using the Wilcoxon rank-sum test [19]. Antibiotic resistance was determined for the 11 agents covering the six antimicrobial categories, namely aminoglycosides, cephems, carbapenems, penicillins, folate pathway inhibitors, and tetracyclines. Among the 154 test isolates, only 6.4% were not biofilm producers, 15.6% were weak biofilm formers, 32.4% (50 isolates) were moderate biofilm formers, and 45.4% (70 isolates) were strong biofilm formers (Table 2). 

**Figure 2:**
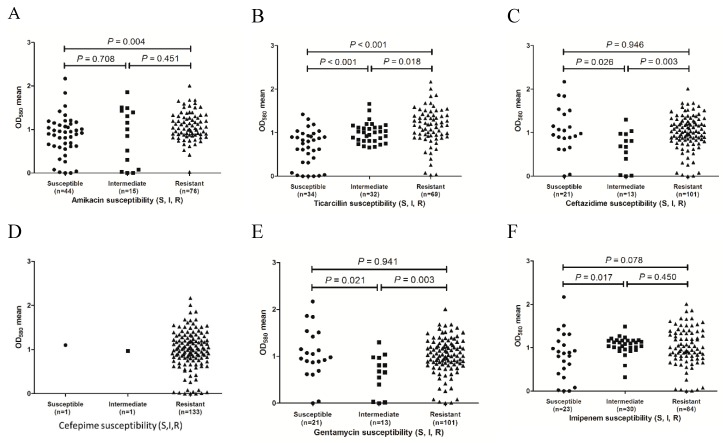

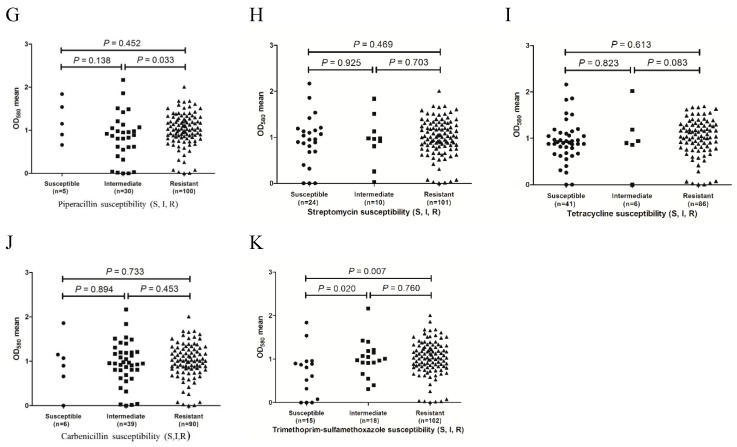
Distribution of biofilm formation of the isolates with different antibiotic resistance phenotypes. (**A**), amikacin susceptibility; (**B**), ticarcillin susceptibility; (**C**), ceftazidime susceptibility; (**D**), cefepime susceptibility; (**E**), gentamicin susceptibility; (**F**), imipenem susceptibility; (**G**), piperacillin susceptibility; (**H**), streptomycin susceptibility; (**I**), tetracycline susceptibility; (**J**), carbenicillin susceptibility and (**K**), sulfamethoxazole/trimethoprim susceptibility.

To determine whether biofilm formation is correlated with any particular antibiotic resistance, biofilm formers with different resistance profiles for the 11 antibiotics were compared. As shown in Figure 2, the results revealed that for the ticarcillin (Figure 2B), ceftazidime (Figure 2C), gentamicin (Figure 2E), and piperacillin (Figure 2G) antibiotics, the resistant isolates tended to form stronger biofilms than the intermediate isolates (*p* = 0.018, 0.003, 0.003, and 0.033, respectively). For the ticarcillin (Figure 2B), imipenem (Figure 2F), and sulfamethoxazole-trimethoprim (Figure 2K) antibiotics, the susceptible isolates tended to form weaker biofilms than the intermediate isolates (*p* < 0.001, 0.017, and 0.020, respectively). In addition, the isolates with resistance to amikacin (Figure 2A), ticarcillin (Figure 2B), and sulfamethoxazole-trimethoprim (Figure 2K) exhibited stronger biofilm formation than the susceptible isolates (*p* = 0.004, *p* < 0.001, and *p* = 0.007, respectively). The results indicate a positive correlation between biofilm formation capacity and resistance to amikacin, ticarcillin, ceftazidime, gentamicin, piperacillin, imipenem, and sulfamethoxazole-trimethoprim antibiotics. For four out of the 11 antibiotics tested (cephalexin, Figure 2D; streptomycin, Figure 2H; tetracycline, Figure 2I and carbenicillin, Figure 2J), no significant difference in biofilm formation was observed between susceptible and resistant isolates (*p* > 0.05). Due to the substantial differences in sample size, only one isolate was susceptible with immediate resistance to cephalexin, six isolates had immediate resistance to tetracycline, and six isolates were susceptible to carbenicillin. The results might not be confirmed by statistical analysis.

### 2.4. Relationship of Biofilm Formation and the Biofilm Related Genes

The distribution of virulence genes (*bap*, *bla*_PER_, *omp*A, and *csu*E) are involved in the biofilm formation of clinical *A. baumannii* isolates with multidrug resistance [6,7,8,9,10,11,12]. In this study, a polymerase chain reaction was used to determine the presence of biofilm-related genes. The prevalence of *bap*, *bla*_PER_, *omp*A, and *csu*E genes among the test isolates was 79.2%, 38.3%, 91.6%, and 68.8%, respectively (Table 2). Among the 154 test strains, a total of 144 isolates were biofilm formers, of which 45.4% were strong biofilm formers, 32.5% were moderate biofilm formers, and 15.6% were weak biofilm formers (Table 2). After analyzing the association between biofilm formation and biofilm-related genes, the results revealed that the *bap*, *bla*_PER_, *omp*A, and *csu*E genes were found in 81% (116/144), 39% (56/144), 91% (131/144), and 69% (99/144) of the biofilm producers, respectively. As shown in Table 2, the strains carrying *bap*, *bla*_PER_, *omp*A, and *csu*E genes tend to form stronger biofilm than the isolates without these genes.

### 2.5. Microscopic Analysis of Biofilms Formation Ability

**Figure 3:**
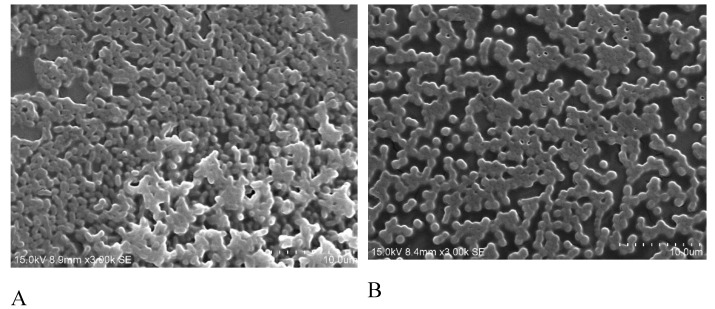
Scanning electron micrograph images of *A. baumannii* biofilm on the surface of minimum biofilm eliminating concentration (MBEC) device. (**A**), strong biofilm formation; (**B**), moderate biofilm formation. Magnification 3000×; Bars = 10 μm.

**Figure 4:**
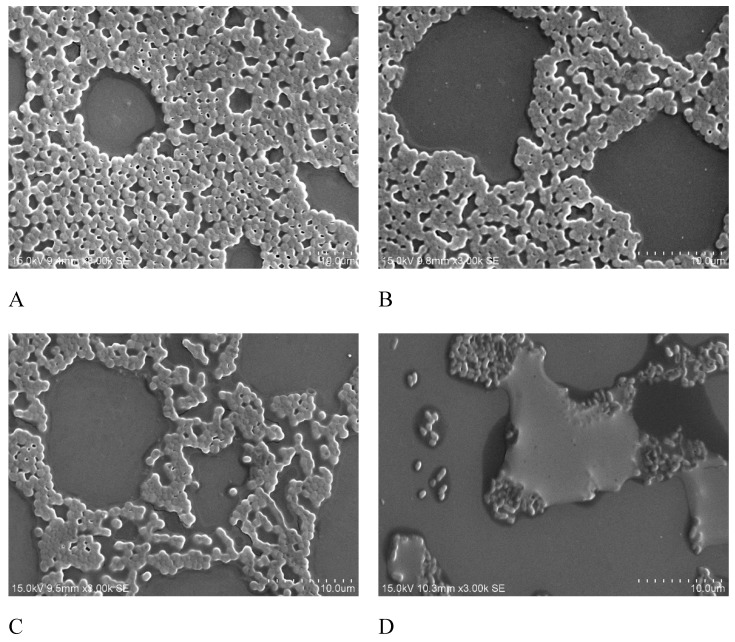
Scanning electron microscopy (SEM) images of *A. baumannii* treated with imipenem. (**A**), After 4 hr of treatment with 16 µg/mL imipenem; (**B**), After 4 hr of treatment with 32 µg/mL imipenem; (**C**), After 8 hr of treatment with 16 µg/mL imipenem; (**D**), After 8 hr of treatment with 32 µg/mL imipenem. Magnification 3000×; Bars = 10 μm.

Biofilm formation on the minimum biofilm eliminating concentration (MBEC) device was observed using scanning electron microscopy (SEM). The SEM analysis revealed that in the moderate-biofilm-forming strains, only a few of the cells were clustered together, whereas in the strong-biofilm-forming strains, large groups of conglomerate cells were found (Figure 3). To analyze the effects of antibiotics on biofilm formation, the isolates were treated with different doses of imipenem and different growing times. SEM images indicated that the biofilm formation is related to treatment time and antibiotics dosage. As shown in Figure 4, the biofilm was clearly inhibited at a higher concentration of imipenem (64 µg/mL) and after longer treatment (8 hr).

## 3. Discussion

*Acinetobacter baumannii*, recently as an increasingly common pathogen, is closely associated with hospital acquired infection [1,2]. Many studies have found that the strong survival ability of *A. baumannii* in strict environments and highly resistant to various antibiotics is due to biofilm formation [3,4,5,6]. The present study investigated relationships among antibiotic resistance, biofilm formation, and the related genes in the clinical isolates of *A. baumannii*. A phenotype profile was compared with biofilm formation and antibiotic resistance, and we observed that antibiotic resistance was highly associated with the biofilm formation capacities. Some of the antibiotic resistant strains had higher biofilm formation capacities under certain antibiotics; for example, penicillin-resistant strains exhibited a greater biofilm formation capacity [20]. That might be due to a constitute stress such as antibiotics that will enhance for induced gene regulation and offer fitness advantages for resistant strains, resulting in biofilm formation [21]. The results suggested that penicillin resistance had a positive correlation with biofilm formation capacity. 

Biofilm formation and antibiotic resistance levels may vary among sites and the key factors responsible for this resistance may differ. Regarding resistance, the primary evidence indicates that conventional mechanisms cannot explain the high resistance to antibacterial agents associated with biofilms [22]. Several mechanisms considered key factors in the high resistance of biofilms have been explored: (a) limited diffusion, (b) enzyme-caused neutralizations, (c) heterogeneous function, (d) slow growth rate, (e) persistent (nondividing) cells, and (f) biofilm phenotype adaptive mechanisms [22,23].

Agar-based antibacterial susceptibility testing, such as the disk diffusion method, has a lower cost and less labor compared with the broth dilution method. In addition, the disk diffusion assay only provides a zone of inhibition and does not generate a minimum inhibitory concentration (MIC) for each antibiotics tested. Thus, according to the results obtained from the disk diffusion test, we selected 75 *A. baumannii* isolates for the MIC determination by the broth dilution assay. Some recent studies have reported that exposure of strains to MICs of certain antibiotics promotes biofilm formation, indicating that biofilms tend to be more robust when antibiotic resistance is challenged [22,23,24], this is consistent with the results of the present study. In addition, our study found that the strong biofilm producers tended to be resistant against numerous antibiotics, including ticarcillin, ceftazidime, gentamicin, and piperacillin. Among these antibiotics, ticarcillin and piperacillin belong to penicillin antibiotics and their robust biofilm formation is associated with antibiotics in the penicillin class, as reported in the previous research [24]. However, in the present study, we found that resistance to aminoglycoside antibiotics was also related to biofilm formation; this has not been reported in any previous studies. We postulate that this may be because aminoglycosides are frequently ineffective against strains of *A. baumannii*, and thus combinations of aminoglycosides and carbapenems are often applied to yield synergistic effects for treatment of infected patients in hospitals [25]. Therefore, the positive correlation between aminoglycoside resistance and biofilm formation could be due to the synergistic effects of both antibiotics.

Although no studies have reported a relationship between aminoglycoside resistance and biofilm formation in *A. baumannii*, Hoffman et al. observed that aminoglycoside antibiotics induced biofilm formation in *P*. *aeruginosa* and *Escherichia coli* [26]. In *P. aeruginosa*, a gene, namely aminoglycoside response regulator (*arr*), was essential for induction and contributed to biofilm-specific aminoglycoside resistance. In the present study, based on the results of antibiotic susceptibility tests, aminoglycoside antibiotics induced bacterial biofilm formation in *A. baumannii*. In addition to the correlation between antibiotic resistance and biofilm formation, the relationship between biofilm formation and related genes, including *bap*, *csu*E, *omp*A and *bla*_PER-1_, were evaluated in this study. The biofilm associated protein is expressed on the cell surfaces of bacteria; many of the *bap* gene carriers of *A. baumannii* exhibit biofilm production on both biotic and abiotic surfaces [7,8]. In the study, molecular analyses showed that 122 (79.2%) clinical isolates of *A. baumannii* harbored the *bap* gene. In addition, the statistical analysis revealed that the emergence of *bap* and biofilm formation was related to the connection. The biofilm related gene, *csu*E, is a member of the usher-chaperone assembly system, which mediate attachment and biofilm formation. In the present study, the *csu*E gene harboring strains accounted for 68.8% of the test isolates.

In 2008, Lee et al suggested that biofilm formation in *A. baumannii* was related to the *bla*_PER-1_ gene [11]. *A. baumannii* individuals harboring the extended-spectrum-resistant gene *bla*_PER-1_ formed a considerably higher biofilm formation than those that lacked *bla*_PER-1_ [27,28]. In the present study, the prevalence of the *bla*_PER-1_ gene was 38.3% in the test strains. However, one study [29] reported no relationship between biofilm formation and production of *PER*-1 β-lactamase. Therefore, a possible explanation for the striking characteristic of *A. baumannii* could be that *bla*_PER-1_ increases the adhesion of cells that carry the gene without necessarily contributing to biofilm formation.

Among the outer membrane proteins identified in *A*. *baumannii*, Ab*Omp*A (*Omp*A) is the most abundant surface protein [23]. Ab*Omp*A, acts as a porin, is required for eukaryotic cell adhesion, and partially contributes to serum resistance and biofilm formation [30]. The *Omp*A harboring strains accounted for 91.6% of the strains in the current study, and some of the non-biofilm-forming strains also contained the *Omp*A gene. However, no further evidence is available to ascertain whether *Omp*A induces biofilm formation. 

Scanning electron microscopy (SEM) is a useful tool for investigating surface structures of biological samples [31]. In a SEM observation, *A*. *baumannii* cells were connected to one another with extracellular appendages [31]. Imipenem, a subgroup of carbapenems antibiotics, has a broad spectrum of activity against aerobic and anaerobic Gram positive as well as Gram negative bacteria. Many previous studies demonstrated that imipenem was highly effective against biofilm formation [31,32]. Thus, we used imipenem to determine the effect of antibiotics treatment on biofilm formations. In the present study, we conducted the SEM observation to determine the effect of imipenem treatment on the surface structures of biofilms grown on Minimum Biofilm Eradication Concentration (MBEC) pegs. No studies have reported the correlation between genotypes and adherence by prokaryotic cells [33]. The SEM diagrams revealed the role of imipenem on biofilm production, although the mechanism has not yet been clearly elucidated. The quantitative differences in biofilm formation among clinical isolates and their relationships with the epidemicity of strains and severity of infections have been poorly investigated, and thus such critical aspects require further study [34]. 

The experimental results were analyzed through statistical methods and revealed that biofilm formation is associated with the following five antibiotics: Tetracycline, sulfamethoxazole-triethoprim, gentamicin, ceftazidime, and ticarcillin. These five antibiotics, commonly used in hospitals, are categorized into five types: Tetracycline, folate pathway inhibitors, aminoglycosides, carbapenems, and penicillins. Based on the selection of antibiotics, biofilm formation by pathogens exhibits varying performance. Although not every antibiotic is associated with stronger biofilm formation, statistical analyses have revealed that biofilm formation is related to a strain’s susceptibility to an antibiotic.

## 4. Material and Methods

### 4.1. Bacterial Strains 

A total of 154 antibiotic resistant strains of *Acinetobacter baumannii* were isolated from Chiayi Christian Hospital (Chiayi, Taiwan). All strains were stored at –80 °C, and bacteria were grown overnight at 37 °C on Mueller-Hinton agar (MHA). Standard strain used in this study was *Acinetobacter baumannii* ATCC19606.

### 4.2. Antibiotic Susceptibility Test

The antibiotic susceptibility of *Acinetobacter baumannii* isolates are based on the results of disc diffusion and minimum inhibitory concentration (MIC). The disk diffusion method is according to CLSI guidelines [16]. Eleven different antibiotics were used to assess the susceptibility test including imipenem (10 µg), cefepime, (30 µg), ceftazidime (30 µg), amikacin (30 µg), gentamicin (10 µg), tetracycline (30 µg), ticarcillin (75 µg), piperacillin (100 mg), sulfamethoxazole/trimethoprim (25 µg), carbenicillin (100 µg) and streptomycin (10 µg) (Sigma-Aldrich, St. Louis, MI, USA).

Broth dilution method was used to determine the minimum inhibitory concentration according to CLSI guidelines [16]. The antibiotics imipenem, cefepime, ceftazidime, amikacin, gentamicin, tetracycline, ticarcillin, piperacillin, sulfamethoxazole/trimethoprim, carbenicillin and streptomycin (Sigma-Aldrich) were used for MIC determination. Multidrug resistance was defined in this analysis as resistance following five drug classes: Extended-spectrum cephalosporins (ceftazidime and cefepime), beta lactamase inhibitor penicillin (Ticarcillin, Piperacillin and Carbenicillin), aminoglycosides (amikacin, gentamicin and streptomycin), Folate pathway inhibitors (sulfamethoxazole/trimethoprim) and carbapenems (imipenem). 

### 4.3. Detection of Biofilm Related Genes 

Polymerase chain reaction (PCR) assays for detection of *bap*, *bla*PER-1, *csu*E and *omp*A genes were performed by a set of primers as shown in Table 3 [29,32,35]. DNA was extracted from each isolate by genomic DNA extraction kit (Geneaid, Taiwan). PCR assays were performed using PCR Red Master Mix (AMPLIQON, Paris, France) in an ABI thermo cycler (Applied Biosystems 2720, Foster City, CA, USA). PCRs were carried out in 25 µL reaction volume and consisted of 5 µL of genomic DNA (5 ng), 12.5 µL PCR Master Mix, 2.0 U of *Taq* DNA polymerase, 10 mM dNTP mix at a final concentration of 0.2 mM, 50 mM MgCl_2_ at a final concentration of 1.5 mM, 1 mM of each primer, 1X PCR buffer (final concentration) and 1 µL (10 pmol) of each primer. Conditions for the PCR were initial denaturation at 94 ℃ for 5 min, followed by 35 cycles of denaturation at 94 ℃ for 60 s, an annealing temperature for each gene (according to Table 1) for 1 min, an extension at 72 ℃ for 45 s, followed by a final extension at 72 ℃ for 5 min. Positive and negative controls were included in all PCR assays. 

### 4.4. Quantitative Biofilm Formation Assay

The biofilm formation ability of *A. baumannii* isolates was determined by polystyrene tube assay based on the crystal violet staining method [33]. Briefly, polystyrene (12 mm × 75 mm) tubes containing 1.5 ml of Mueller–Hinton broth were inoculated with 30 μL of an overnight liquid culture with OD_600_ = 0.1, and the tubes were incubated at 37 °C for 48 h. The liquid media was discarded, and the adherent cells were washed twice with phosphate-buffered saline (PBS) and stained with 0.02% of crystal violet for 10 min. The stain was eluted from the adherent cells using an ethanol solvent and vortexing for 5 min. Absorbance of the eluted solvent was measured, after diluting 10-fold with the solvent, at 580 nm using an UV visible spectrophotometer (Shishin, SH-U830, Taipei, Taiwan, ROC). The assay was done at least three times using fresh samples each time.

The optical density cut-off value (ODc) was established as three standard deviations (SD) above the mean of the optical density (OD) of the negative control as shown in the following formula: ODc = average OD of negative control + (3 × SD of negative control). The results were divided into four categories according to their optical densities as (1) strong biofilm producer (4 × ODc < OD); (2) medium biofilm producer (2 × ODc < OD ≤ 4 × ODc); (3) weak biofilm producer (ODc < OD ≤ 2 × ODc); and (4) non-biofilm producer (OD ≤ ODc) [29]. 

### 4.5. Microscopic Analysis of Biofilms Formation Ability

The biofilm formation ability of *A. baumannii* strains was visualized by scanning electron microscope (SEM) (Hitachi-S3400, Tokyo, Japan). Biofilm was formed on the minimum biofilm eliminating concentration device (MBEC™ P&G Physiology & Genetics Innovotech, Alberta, Canada). Briefly, *A. baumannii* suspensions (200 μL) were inoculated into each well and then incubated overnight at 37 °C. Biofilms that formed were then washed twice with PBS to remove any unattached and floating cells and were fixed with 2.5% glutaraldehyde in 0.1 M cacodylic acid (pH 7.2) at 4 °C for 24 h and post fixed with 0.1 M cacodylic acid for approximately 10 min. After incubation, the plates were washed twice with distilled water for 15 min, followed by gradual dehydration with ethanol, and air dry for a minimum of 24 h. The fixed biofilms were then coated with a layer of gold–palladium (7 nm thick) and examined with SEM (Hitachi-S3400) [36]. 

### 4.6. Statistical Analyses 

The relationship between biofilm formation and antibiotic susceptibility was analyzed by Wilcoxon rank sum test. All analyses were carried out with one-way ANOVA. Categorical variables between more than two groups were tested, and *P* values of ≤ 0.05 indicated statistical significance.

## 5. Conclusions

In this study, the molecular genotypes and phenotypes of clinical antibiotic-resistant *A. baumannii* were investigated, and the correlations among antibiotic resistance, biofilm formation, and biofilm related genes were determined. Our results indicated that the *omp*A and *bap* genes influence biofilm formation and antibiotic resistance patterns based on the statistical analysis. Such mechanisms may facilitate our understanding of the relationship between biofilm production and antibiotic resistance in *A. baumannii*, and that of the routes of transmission of clinical isolates. The relationship between biofilm formation and antibiotic resistance may further provide information that could facilitate attempts to control drug-resistant pathogens.

## Figures and Tables

**Table 1:** Minimum inhibitory concentration (MIC) determination in *A. baumannii* clinical isolates.

Antimicrobial Category	Antimicrobial Agent	Antibiotic Resistance Level (%)			MIC (µg/mL)		
		S	I	R	S	I	R
Aminoglycosides	Gentamicin	36%	23%	41%	≤4	4–8	≥16
	Amikacin	35%	39%	27%	≤16	16–32	≥64
	Streptomycin	24%	44%	32%	≤4	4–8	≥16
Cephems	Cefepime	11%	31%	59%	≤4	4–6	≥16
Carbapenems	Ceftazidime	29%	57%	13%	≤8	8–16	≥32
	Imipenem	35%	37%	28%	≤2	2–4	≥8
Penicillins	Ticarcillin	15%	44%	41%	≤16	16–64	≥128
	Piperacillin	15%	43%	43%	≤16	16–64	≥128
	Carbenicillin	6%	37%	56%	≤16	16–32	≥64
Folate pathway inhibitors	Sulfamethoxazole-Triethoprim	31%	5%	63%	≤4	4–38	≥76
Tetracycline	Tetracycline	59%	15%	27%	≤4	4–8	≥16

**Table 2:** Correlation of the biofilm related genes and biofilm formation.

Biofilm Formation *	Isolates /Biofilm Formation %	Biofilm-Related Genes			
		Isolates/Genes %			
		*bap*	*bla*_PER_	*omp*A	*csu*E
Non biofilm	10/6.5	6/3.9	3/1.9	10/6.5	7/4.5
Weak biofilm	24/15.6	18/11.7	6/3.9	22/14.3	16/10.4
Moderate biofilm	50/32.5	36/23.4	19/12.3	45/29.2	33/21.4
Strong biofilm	70/45.4	62/40.3	31/20.1	64/41.6	50/32.5

Total isolates (*n* = 154); * OD_580_: Biofilm formation was quantified by measuring optical absorbance (580 nm) using crystal violet.

**Table 3:** The primers used in this study for detection of biofilm related genes.

Primers	Primer Sequence (5’-3’)	Product Size (bp)	References
*bap*	TGCTGACAGTGACGTAGAACCACA TGCAACTAGTGGAATAGCAGCCCA	184	[35]
*bla*_PER-1_	GCAACTGCTGCAATACTCGG ATGTGCGACCACAGTACCAG	900	[29]
*csu*E	CATCTTCTATTTCGGTCCC CGGTCTGAGCATTGGTAA	168	[32]
*omp*A	GTTAAAGGCGACGTAGACG CCAGTGTTATCTGTGTGACC	578	[32]
